# Compare the Efficacy and Safety of Modified Combined Short and Long Axis Method versus Oblique Axis Method for Right Internal Jugular Vein Catheterization in Adult Patients (The MCSLOA Trial): Study Protocol of a Randomized Controlled Trial

**DOI:** 10.3389/fsurg.2022.725357

**Published:** 2022-04-29

**Authors:** Jia-Xi Tang, Ling Wang, Wei-Qi Nian, Wan-Yan Tang, Xi-Xi Tang, Jing-Yu Xiao, Hong-Liang Liu

**Affiliations:** ^1^ Department of Anesthesiology, Chongqing University Cancer Hospital, Chongqing, China; ^2^ Department of Phase I Clinical Trial Ward, Chongqing University Cancer Hospital, Chongqing, China

**Keywords:** ultrasound, central venous catheterization, internal jugular vein, IJV, modified combined short and long axis, MCSL, oblique axis

## Abstract

**Background:**

Ultrasound-guided internal jugular vein (IJV) catheterization has become a standard procedure as it yields a higher success rate and fewer mechanical complications compared with an anatomical landmark technique. There are several common methods for ultrasound guidance IJV catheterization, such as short-axis out-of-plane, long-axis in-plane and oblique axis in-plane, but these technologies are still developing. It is important to further study the application of different ultrasound-guided IJV puncture techniques and find an effective and safe ultrasound-guided puncture technique.

**Methods:**

A China randomized, open-label, parallel, single center, positive-controlled, non-inferiority clinical trial will evaluate 190 adult patients undergoing elective surgery and need right jugular vein catheterization. Study participants randomized in a 1:1 ratio into control and experimental groups. The control group will take the oblique axis in-plane method for IJV catheterization. The experimental group will take the Modified combined short and long axis method. The primary endpoint of the trial is the rate of one-time successful guidewire insertion without posterior wall puncture (PWP). Secondary endpoints are the number of needle insertion attempts, the total success rate, the procedure time, and mechanical complications.

**Conclusion:**

This randomized controlled trial will evaluate the effectiveness and safety of Modified combined short and long axis method and oblique axis in-plane method for right IJV catheterization in adult patients.

## Introduction

Central venous catheterization (CVC) plays an important role in clinical treatment, including rapid blood transfusion and fluid infusion, measurements of hemodynamic variables, delivery of medications, and nutritional support. Internal jugular vein (IJV) is one of the most common ways to establish central venous access because of its anatomical position and lower infection rate (1, 2). At present, many guidelines recommend the routine use of ultrasound-guided IJV catheter placement to decrease complications and decrease catheter placement attempts (3–8). Therefore, ultrasound-guided IJV puncture catheterization should become a standard operating procedure.

There are many approaches and techniques for ultrasound-guided IJV catheterization (9–14). The short-axis out-of-plane method can clearly see the adjacent relationship between IJV and common carotid artery (CCA), but because the entire needle body is not visible, there is still the possibility of accidental posterior vessel wall puncture and arterial puncture (15). The long-axis in-plane method ensures the view of the entire needle, including tracking of the needle tip, but it has some disadvantages, including not suitable for short-necked patients, and the relationship between the CCA and IJV is usually lost. The puncture needle needs to be inserted into the narrow sound beam. If the puncture deviates from the ultrasound probe, the needle is still not visible, and accidental arterial puncture may occur (9). Phelan M et al. first proposed the oblique axis in-plane (OA-IP) method in 2009, which is an ultrasound guidance method that combines the advantages of the short axis method and the long axis method (16). Oblique axis view provides a longer view of the IJV along with the CCA.

The current clinical research is mainly about the comparative study of short axis method, long axis method, oblique axis method and modified methods of the above methods, in order to seek a safer and more effective ultrasound-guided IJV puncture method. A large number of studies have shown that the oblique axis method has obvious advantages compared to the long axis and the short axis methods, so it is recommended for internal jugular vein catheterization by many scholars (9, 16–20). In our previous research, we proposed a modified combined short and long axis method (MCSL) (11), which combines the advantages of the modified short axis out of plane approach (MSA-OOP) with the short-axis combined long-axis method (CSLA) to accurately locate puncture needle points and visualize the entire needle body across the entire IJV catheterization (10, 13).

To the best of our knowledge, comparative studies between MCSL and OA-IP are still blank. A prospective randomized controlled, evaluator blind, non-inferiority clinical trial was designed to compare the efficacy and safety of MCSL with OA-IP. We hypothesized that the MCSL method is not inferior to the OA-IP method in puncture success rate for right IJV catheter placement in adults.

## Study Design/Methods

### Overview

**Figure 1:**
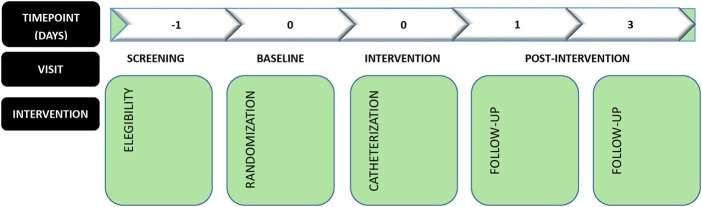
Design overview. Participants will be randomly assigned to either the modified combined short and long axis method or oblique axis in-plane method group.

The modified combined short and long axis method for right IJV catheterization in adult patients is a single-center, open-label, parallel, randomized, positive-controlled, non-inferiority clinical trial. The trial has been approved by the Ethics Committee of Chongqing University Cancer Hospital (CZLS2021042-A), and has been prospectively registered at ChiCTR.org.cn (ChiCTR2100046899) on 30 May 2021. A total of 190 participants will be recruited and randomized to treatment groups for the study. Eligible participants will be randomized to receive one of two methods (1) MCSL, (2) OA-IP for right IJV catheterization and followed for a total of 3 day. During the 3-day trial, participants will be evaluated for outcomes on puncture indicators, ultrasound anatomy and safety risk. Study assessments will be conducted at days 0, 1, and 3 post-randomization (Figure 1).

The trial will be conducted in the Department of Anesthesiology, Chongqing University Cancer Hospital. The randomization will be conducted by biostatistician who is not involved in the study. The random information will be sealed in an opaque envelope till the patient enter the operating room. Participant safety will be supervised by a comprehensive study team—including the principal investigator, study clinicians, study staff, and an appointed Data Safety Monitoring Board. Written informed consent approved by the Institutional Review Board will be obtained from each patient before enrollment.

### Participants

One hundred and ninety persons between the ages of 18 and 75 years old scheduled to undergo elective surgery requiring central vein catheterization at the right internal jugular vein will be eligible to enter the study. Participant inclusion and exclusion criteria are presented in Table 1.

**Table 1:** Inclusion and Exclusion criteria for the MCSLOA trial.

**Inclusion criteria**
18–75 years old
Patients who need right IJV catheterization for elective surgery
**Exclusion criteria**
Failure to provide consent
Abnormal blood coagulation function
INR >1.5, platelet count <50.000
Possible structural abnormalities in the neck
History of previous surgical intervention or radiotherapy near the cannulation site Recent cervical trauma with present neck immobilization Patients with huge masses or lymph nodes in the right neck Infection signs, neck scar, subcutaneous emphysema or subcutaneous haematoma close to the puncture site History of right IJV catheterization during the past 1 month
Contraindications of internal jugular venipuncture
Presence of Superior vena cava syndrome currently IJV plaque thrombosis Patients with anatomical variations and no right IJV
Right chest surgery
Agitated or uncooperative patient

*IJV, internal jugular vein; INR, international standard ratio.*

### Screening and Randomization

At the screening visit before surgery, participants sign the informed consent form and are assessed for study eligibility by investigator.

**Figure 2:**
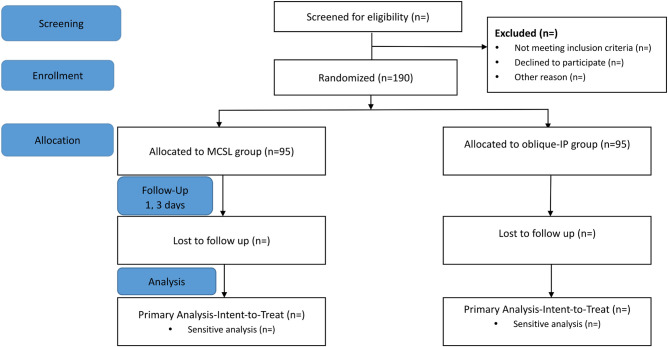
Study Design in Consolidated Standards of Reporting Trials (CONSORT) Format. Due to safety, mechanical complications such as pneumothorax, hemothorax, and hematoma will be followed up for 3 days.

Participants who meet all eligibility criteria will be performed a baseline assessment and randomized (Figure 2). Blocks of 4 randomization is used to assign participants to intervention arms, with 1:1 allocation ratio, to ensure approximately equal accrual to each intervention group throughout the study. Computer-generated randomization sequence will be created and maintained by the study biostatistician. Opaque envelopes will be used to maintain allocation concealment. Because of the specificity of the puncture procedure, patients and investigators will not be blinded to group assignment. Data monitors and statistical analysts will be blinded in this study.

### Intervention

All patients will be kept nil per oral, as per standard guidelines and their hydration status was maintained with continuous intravenous hydration with Isolate-P™. All CVC procedures will be performed by the same attending physician (Tang) who has completed ultrasound-guided internal jugular vein puncture for more than 500 cases. All patients will be anaesthetized with a standard general anesthetic technique, and the airway will be secured by tracheal intubation before positioning them for CVC. After anesthesia, the patient lies in a supine position, with a thin pillow under the shoulder to extend the neck, tilts the head to the left 30 degrees, and lowers the head to 15 degrees. IJV catheterization will be performed using the Seldinger technique. An 18-gauge, 6.35-cm - long needle and a 5-French (16 gauge), 20-cm, single-lumen or dual-lumen catheter (BIOSENSORS INTERNATIONAL™) will be used in all the study cases. All imaging will be done with Mindray UMT-500 portable ultrasound machine (Mindray Inc., Shenzhen, China) using a linear array transducer (frequency of 8–13 Hz, depth of 3.5 cm) with 47 mm footprint.

#### Oblique Axis in-Plane Mthod

Patients in the control group will receive right IJV catheterization using the lateral oblique approach described in the previous study (20). In this group, the ultrasound probe is placed on the jugular vein at mid-neck level to capture a transverse cross-sectional image of the CCA and IJV together. Once the short-axis view is obtained, the probe is rotated 45 degrees clockwise, with the orientation marker medially and the needle will be inserted from the lateral to medial by using in-plane technology.

#### Modified Combined Short and Long Axis Method

For the experimental group, procedures will be conducted as described in our previous report (11). The braided silk suture is tied parallelly (interval: 10 mm) on the ultrasound probe and positions perpendicular to the long axis of the probe to prepare the ultrasound probe before IJV puncture. In this group, the ultrasound probe will be placed in supraclavicular fossa sagittal position to determine the plane of the puncture needle. Then insert the needle between the two lines on the probe at an angle of 30° to the skin with the short-axis out-of-plane technique and visualize the needle tip as a white spot between the two shadows on the ultrasound screen. Rotate the ultrasound probe 90 ◦ clockwise and puncture the anterior wall of the IJV with the long axis in-plane technique.

### Assessment Measures

The primary outcome of the study will be the rate of one-time successful guidewire insertion without posterior wall puncture (PWP). Secondary outcomes include the number of needle insertion attempts, the total success rate, the procedure time (positioning time, guidewire insertion time, total catheterization time), and mechanical complications (posterior vessel wall puncture, carotid artery puncture, hematoma, pneumothorax, and hemothorax). Two assessors (Xiao and Tang XX) who does not perform procedures will record the puncturing procedure on video and assess the aforementioned outcomes. The two assessors had 5 years of point-of-care ultrasound experience and >300 ultrasound-guided vascular access procedures experience. The two assessors independently make the judgments based on the videos. Any disagreement between the two authors will be resolved by a discussion. If there is no consensus, a third assessor (Liu) will be consulted. Extended details on the deﬁnition and measurement of primary and secondary outcomes are provided below. A timetable of events is also provided (Table 2).

**Table 2:** Data collection summary via assessment visit.

	Study Phase				
	Enrollment	Allocation	Intervention	Post-intervention	
Timeline (days)	−1	0	0	1	3
Informed consent	x				
Physical examination, medical history, medication use	x				
Tests (biochemistry, hematology, etc.)	x				
Randomization		x			
Baseline assessment (BMI, neck length, fasting time, ultrasonic anatomical characteristics in IJV)		x			
First time success without PWP rate			x		
Total success rate			x		
procedure time			x		
The number of needle insertion attempts			x		
The rates of PWP			x		
Hematoma			x	x	x
Artery penetration			x		
Pneumothorax			x	x	x
Hemothorax			x	x	x

*BMI, body mass index; IJV, internal jugular vein; PWP, posterior wall puncture.*

#### Primary Outcome

The primary outcome is the rate of one-time successful guidewire insertion without PWP. In ultrasound images, the second wall penetration by the needle after having penetrated the vessel once at the original site of vessel penetration, this is considered a posterior wall penetration (21). During the IJV puncture, the number of patients with one-time successful guidewire insertion without PWP will be recorded in two groups. The proportion of patients achieving the success of the procedure will be calculated.

#### Secondary Outcomes

The number of needle insertion attempts is defined as the number of attempts and redirections of the needle needed for successful catheterization (another puncture attempt is determined by withdrawing and redirecting the needle). Probe placement difficulty is defined as a situation where the placement of the probe significantly affects the penetration. Unsuccessful catheterization is defined as failure to successfully catheterize, including multiple attempts (>3), probe placement difficulty or caused by complications (carotid artery puncture, hematoma, pneumothorax, and hemothorax). The number of needle insertion attempts and procedure time will be recorded as *n* + for patients whose puncture is unsuccessful after more than 3 times, or who are judged to have failed due to mechanical complications. The total success rate is defined as the proportion of successful catheterization with less than 3 times catheterization and no serious complications. Positioning time is defined as the time from probe scan of the blood vessel to the time the puncture is initiated. Guidewire insertion time is defined as the time from the needle insertion until ultrasonic confirmation that the guidewire is in the IJV. Total catheterization time is defined as the time starting from the first skin puncture until the placement of the central venous catheter and unobstructed blood collection. Mechanical complications will be diagnosed by ultrasonography, other imaging examination and clinical evaluation. Carotid artery puncture is defined as the observation of pulsatile blood reflux through the needle or in the ultrasound images, the penetration of a needle into an artery. Hematoma formation is identified by ultrasound. Plain X-ray chest for conﬁrmation of pneumothorax and hemothorax will be a routine practice. Consider that some indicators of complications, such as hematoma, pneumothorax, and hemothorax, may be delayed. For the sake of safety, we set the follow-up time as 1 and 3 days after CVC.

#### Supportive Index Measures

In order to interpret the study outcomes, additional supportive index will be measured. The supportive indexes include body mass index (BMI), neck length, fasting time, ultrasonic anatomical characteristics in IJV (transverse and longitudinal diameter of IJV, distance between IJV and skin, overlap distance between IJV and CCA).

### Safety

Several safety procedures will be used to ensure the safety of participants. First, the exclusion criteria are designed to exclude those with major risk. In order to avoid the injury caused by repeated puncture, it is stipulated that if the puncture is not successful after 3 times, the puncture operation is seriously affected due to the probe placement difficulty or puncture complications occurred, the puncture will be judged as failure, and the central vein catheterization will be completed by replacing the site.

All of the experimental team members have rich experience in ultrasound-guided vascular intervention to ensure the safety of participants. The mechanical complications associated with the intervention will be closely monitored throughout the trial, and possible mechanical complications will be diagnosed and managed by professional clinicians and radiologists. For patients with complications, corresponding treatment will be carried out, and sufficient follow-up will be carried out until the patients fully recover.

### Sample Size Calculation

The sample size calculation for this non-inferiority trial is based on the unpublished data from a Preliminary test of 40 patients. The rate of one-time successful guidewire insertion without PWP of MCSL method is 95%, and the rate of one-time successful guidewire insertion without PWP of OA-IP is 90%. The predeﬁned non-inferiority margin is an absolute difference of 8% in the primary endpoint between groups. With a power of 90%, and a one-sided alpha of 2.5%, a total sample size of 170 (85 per group) will be needed at a 5% signiﬁcance level. We estimate lost-to-follow-up of 10% and therefore increased the sample size by 10% (to 95 subjects per group) to allow for dropouts.

### Statistical Analyses

Efﬁcacy analysis will be performed by intent-to-treat (ITT) set, which consisted of all randomized patients who have received IJV catheterization intervention. All results of efﬁcacy analysis will be analyzed in full analysis set (FAS) and Per-protocol set (PPS), which included all randomized patients without major protocol deviations. Safety assessment will be analyzed in safety analysis set (SS), which consisted of all patients who have received IJV catheterization procedure and have Safety index.

Quantitative data will be presented as the mean ± standard deviation and median (P25–P75) and be analyzed with SPSS software version 21.0. Quantitative data will be compared between groups using Student’s t test or Mann-Whitney U test. Categorical measures will be summarized using counts/frequencies (%) and be compared between groups using χ^2^ analysis or Fisher's exact test. The procedure time will be evaluated using Kaplan-Meier curves and be compared between groups using the log-rank test. Statistical significance will be accepted at *p* < 0.05. For primary outcome, we assessed non-inferiority of the co-primary endpoints with single-sided 95% CIs. Except for analysis of the primary outcome, statistical tests were two-sided, signiﬁcance set at *p* < 0.05 along with 95% conﬁdence intervals (CI).

## Discussion

There is a consensus that ultrasound guidance is mandatory for IJV catheterization procedures, which clearly reduces the number of complications, failures and time required for insertion compared to the anatomic landmark technique (3–8). There are many approaches and techniques for ultrasound-guided central venous catheterization (9, 15, 22). Which ultrasonic guidance method has more advantages has become a research hotspot.

The oblique axis in-plane (OA-IP) method was first proposed by Phelan M et al. in 2009, which is an ultrasound guidance method that combines the advantages of the short axis method and the long axis method (16), and allows simultaneous visualization of both artery and vein and also can optimize needle visualization. This method can increase the confidence of the puncture operators and reduce the complications, especially the posterior wall penetration and accidental arterial puncture (9, 17).

A novel modified short axis out of plane approach (MSA-OOP) for ultrasound-guided IJV cannulation in infants was recently described. In the method, a radiopaque barium wire was used in the midpoint of the ultrasound probe to guide the puncture needle to choose the puncture position (13)^.^ Akihito Tampo first proposed the short-axis combined long-axis (CSLA) technique (23). Like the oblique axis method, the CSLA method also combines the advantages of the long-axis and short-axis methods. Integrating the MSA-OOP and CSLA, we propose a modified combined short and long axis method (MCSL) (11). Our method not only can quickly locate the entry position of the puncture needle, but also can see the whole puncture needle body during the catheterization procedure. In order to reduce the difficulty of probe placement in patients with short neck puncture using MCSL method, the long axis length of ultrasonic probe used in this study is 4.7 mm, which can minimize the occurrence of this situation. We hypothesize that MCSL could be used safely and effectively for internal jugular vein puncture in adult patient.

This is the first time to compare the efficacy and safety between OA-IP method and MCSL method for IJV catheterization. This study design was informed by several successes and challenges encountered as lessons learned during the pilot study phase. Brieﬂy, changes from the pilot phase to facilitate success include: (1) the inclusion criteria were changed from the previous BMI > 28 kg/m^2^ to no restriction on BMI index. (2) The evaluators were changed to two investigators who were not involved in the procedure, and the two evaluators separately evaluated the study metrics based on the puncture video. Ultimately, this project has potential implications for inﬂuencing clinical practice guidelines in IJV catheterization of adults.
